# Supplementary material for: Understanding the Role of Technology Anxiety in the Adoption of Digital Health Technologies (DHTs) by Older Adults with Chronic Diseases in Shanghai: An Extension of the Unified Theory of Acceptance and Use of Technology (UTAUT) Model
Source: Healthcare (Basel). 2024 Jul 16;12(14):1421. doi: 10.3390/healthcare12141421 (PMC11275594; doi:10.3390/healthcare12141421)

Supplemental Figure S1
The illustrative instruction of the applications of digital health technologies (English version)

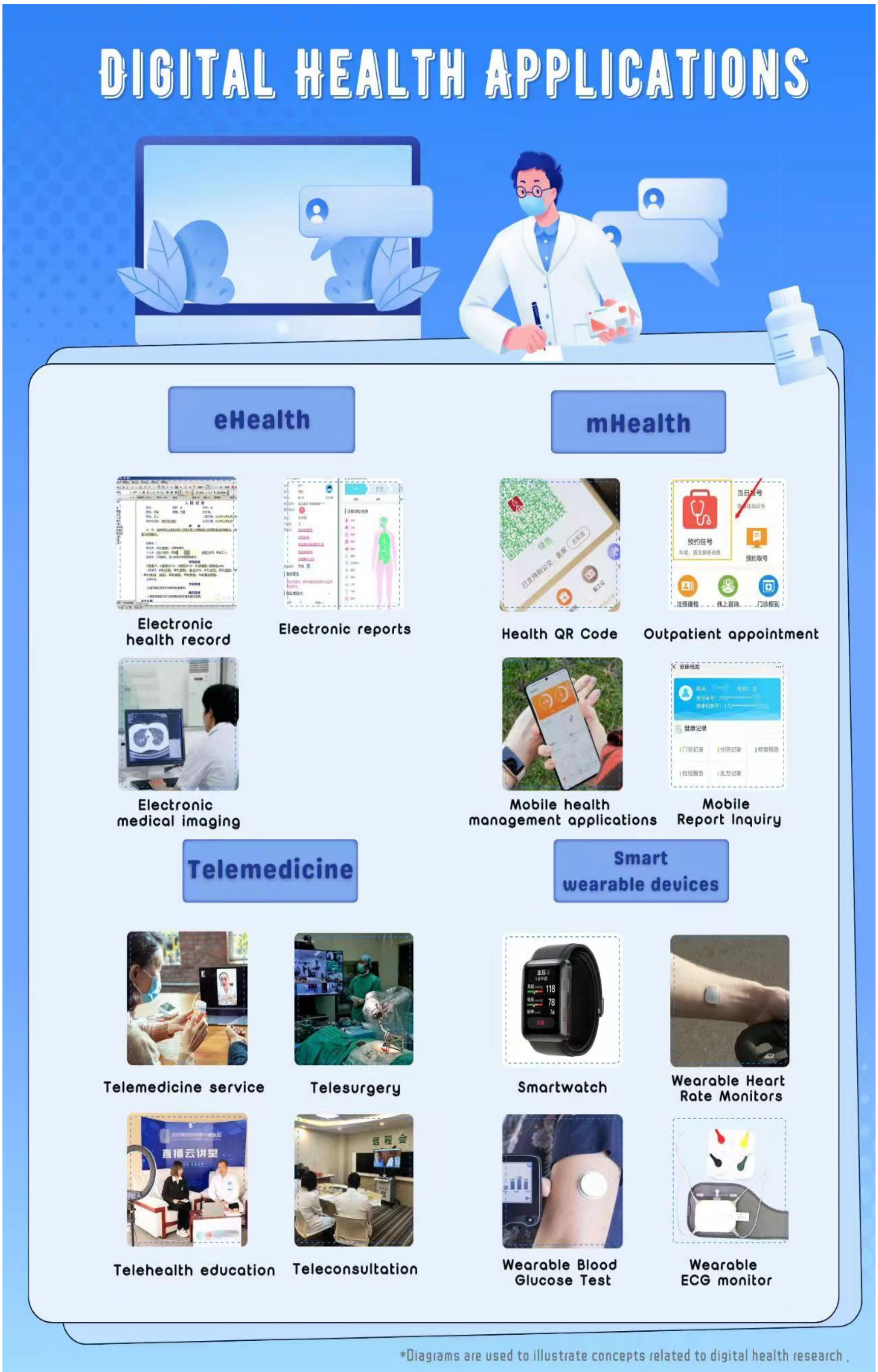

Supplemental Figure S2

The illustrative instruction of the applications of digital health technologies (Chinese version)

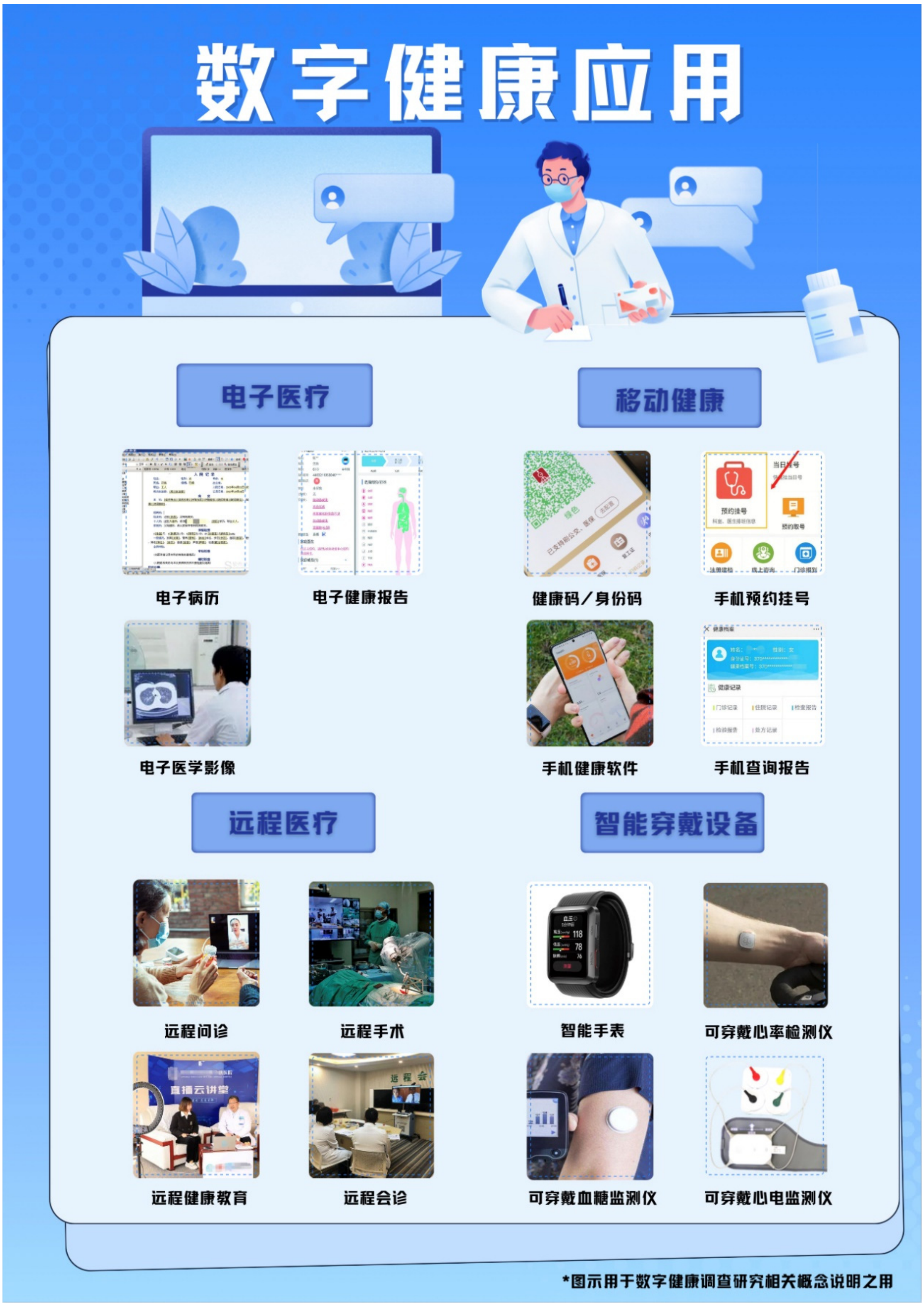

Supplement: Supplementary file 1 [file healthcare-12-01421-s001.zip › healthcare-3073604-supplementary.pdf]
